# GSK-3β deletion in dentate gyrus excitatory neuron impairs synaptic plasticity and memory

**DOI:** 10.1038/s41598-017-06173-4

**Published:** 2017-07-18

**Authors:** Enjie Liu, Ao-Ji Xie, Qiuzhi Zhou, Mengzhu Li, Shujuan Zhang, Shihong Li, Weijin Wang, Xiaochuan Wang, Qun Wang, Jian-Zhi Wang

**Affiliations:** 1 Department of Pathophysiology, School of Basic Medicine and the Collaborative Innovation Center for Brain Science, Key Laboratory of Ministry of Education of China for Neurological Disorders, Tongji Medical College, Huazhong University of Science and Technology, Wuhan, 430030 PR China; 2 Co-innovation Center of Neuroregeneration, Nantong, 226000 PR China

## Abstract

Increasing evidence suggests that glycogen synthase kinase-3β (GSK-3β) plays a crucial role in neurodegenerative/psychiatric disorders, while pan-neural knockout of GSK-3β also shows detrimental effects. Currently, the function of GSK-3β in specific type of neurons is elusive. Here, we infused AAV-CaMKII-Cre-2A-eGFP into GSK-3β^lox/lox^ mice to selectively delete the kinase in excitatory neurons of hippocampal dentate gyrus (DG), and studied the effects on cognitive/psychiatric behaviors and the molecular mechanisms. We found that mice with GSK-3β deletion in DG excitatory neurons displayed spatial and fear memory defects with an anti-anxiety behavior. Further studies demonstrated that GSK-3β deletion in DG subset inhibited hippocampal synaptic transmission and reduced levels of GluN1, GluN2A and GluN2B (NMDAR subunits), GluA1 (AMPAR subunit), PSD93 and drebrin (postsynaptic structural proteins), and synaptophysin (presynaptic protein). GSK-3β deletion also suppressed the activity-dependent neural activation and calcium/calmodulin-dependent protein kinase II (CaMKII)/CaMKIV-cAMP response element binding protein (CREB) signaling. Our data suggest that GSK-3β in hippocampal DG excitatory neurons is essential for maintaining synaptic plasticity and memory.

## Introduction

Glycogen synthase kinase-3 (GSK-3) is highly expressed in central nervous system. Of the two isoforms of GSK-3, i.e. GSK-3α and GSK-3β, activation of GSK-3β is involved in neurodegenerative and psychiatric disorders^1–3^. For instance, elevation of the active form of GSK-3β has been shown in tangle-bearing neurons of the patients with Alzheimer’s disease (AD)^4^. Both *in vitro* and *in vivo* studies demonstrate that upregulation of GSK-3β induces AD-like hyperphosphorylation of microtubule-associated protein tau and causes memory deficit, while inhibition of GSK-3 attenuates tau hyperphosphorylation with improvement of cognitive synaptic functions^5–10^. Thus inhibition of GSK-3β has been considered as a potential therapeutic target of neurodegenerative and psychiatric disorders^9, 11–15^.

However, GSK-3β has diverse physiological functions related to neurogenesis, intracellular signaling, synaptic plasticity and neuronal viabilities^16–19^. In hippocampus, GSK-3β is required for induction of long term depression (LTD)^20^. Activation of GSK-3β enhances spontaneous firing rate (SFR)^21^ and contributes to neuronal excitability^22^. GSK-3β knockout causes embryonic lethality in murine^23^, and global heterozygous GSK-3β knockout in mice induces long-term spatial memory impairment^24^. In neuronal levels, loss of GSK-3β reduces dendritic spine stability with an impaired synaptic transmission in cortices and hippocampal CA1 subset^25^. These data suggest that global knockout of GSK-3β also causes negative effects.

The trisynaptic circuit in hippocampus formation (i.e. entorhinal cortex (EC) to dentate gyrus (DG)/DG to CA3/CA3 to CA1) is essential for spatial memory and emotional behaviors^26–29^. The excitatory neuron in DG is crucial for encoding of contextual information^30–32^. Although a previous study has shown that pan-neuronal silence of GSK-3β in adult DG impairs contextual fear memory with an enhanced LTP^33^, the function of GSK-3β in DG excitatory neuron is not reported.

In the present study, we designed an adenovirus-associated virus vector (AAV-CaMKII-Cre-2A-eGFP) to specifically delete GSK-3β in hippocampal DG excitatory neurons of GSK-3β floxed mice (GSK-3β^lox/lox^)^34^. We found that GSK-3β deletion in hippocampal DG excitatory neurons induced spatial and fear memory deficits with an anti-anxiety behavior. The molecular mechanism involves CaMKII/CaMKIV/CREB signaling-associated synaptic impairments.

## Results

### Deletion of GSK-3β in DG excitatory neurons in GSK-3β^lox/lox^ mice

**Figure 1:**
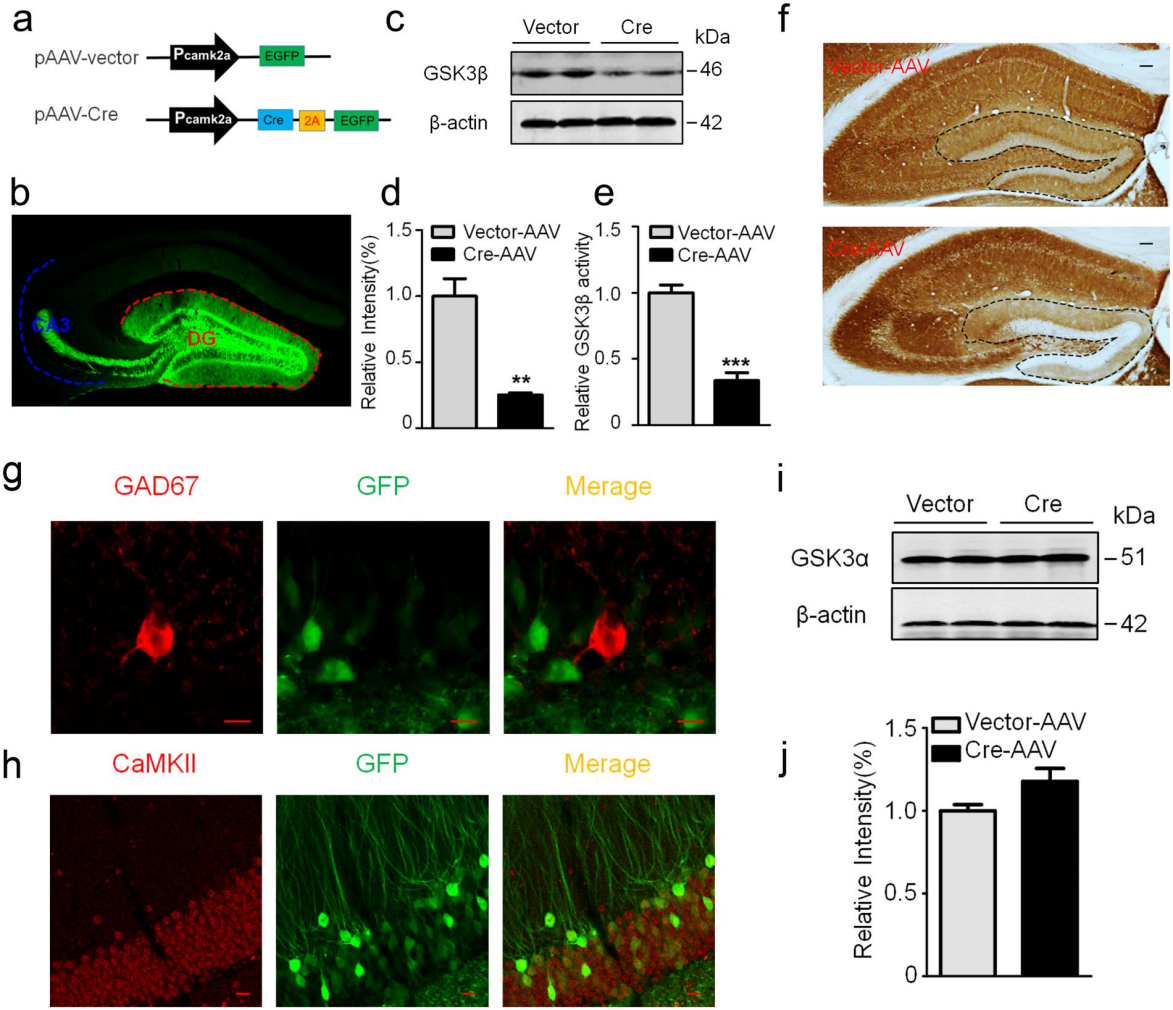
Expression of Cre recombinase selectively deletes GSK-3β in DG excitatory neurons of GSK-3β loxp mice. (**a**) Schematic representation of pAAV-CaMKII-Cre-2A-eGFP and the empty pAAV-CaMKII-eGFP vector. (**b**) A representative image showing efficient virus infection in DG and the mossy fibers. (**c,d**) Injection of Cre recombinase into hippocampal DG of GSK-3β loxp mice for 1-m efficiently downregulated GSK-3β protein level measured by Western blotting (n = 3 each group). (**e**) The reduced GSK-3β activity in DG extracts after Cre recombinase injection (n = 3 each group). (**f**) Deletion of GSK-3β in DG neurons by Cre recombinase injection measured by immunohistochemistry, scale bars, 100 μm. (**g,h**) Specific deletion of GSK-3β in DG excitatory neurons measured by co-immunofluorescence of GFP with CaMKII but not with GAD67 antibody, scale bars, 10 μm. (**i**,**j**) Deletion of GSK-3β did not significantly affect GSK-3α in DG subset (n = 3 each group). Data were presented as mean ± s.e.m. unpaired t test, **P < 0.01, ***P < 0.001 *versus* Vector. The absence of asterix indicates that the difference is not significant.

To investigate the function of GSK-3β in DG excitatory neurons, we first infused stereotaxically AAV-CaMKII-Cre-2A-eGFP (Cre recombinase) or its empty vector (as control) (Fig. 1a) into the hippocampal DG subset of GSK-3β^lox/lox^ mice to induce a specific deletion of GSK-3β in DG excitatory neurons. The expression of the virus vector in hippocampal DG and the Mossy fibers was detected by direct fluorescence imaging at 4 weeks after infusion of the Cre recombinase (Fig. 1b, Supplementary Fig. 1). A significant reduction of GSK-3β protein level was confirmed by Western blotting (Fig. 1c,d) with a remarkable decrease of GSK-3β activity in DG subset (Fig. 1e). Immunohistochemistry data showed that expression of Cre recombinase almost completely deleted GSK-3β expression in hippocampal DG (Fig. 1f, Supplementary Fig. 2), and specific deletion of GSK-3β in DG excitatory neurons was confirmed by co-immunofluorescence staining of GFP with CaMKII but not GAD67 (Fig. 1g,h). No significant change of GSK-3α protein level was detected (Fig. 1i,j). These data demonstrate an effective and specific deletion of GSK-3β in hippocampal DG of GSK-3β^lox/lox^ mice by Cre recombinase.

### GSK-3β deletion in DG excitatory neurons induces spatial and fear memory deficits

**Figure 2:**
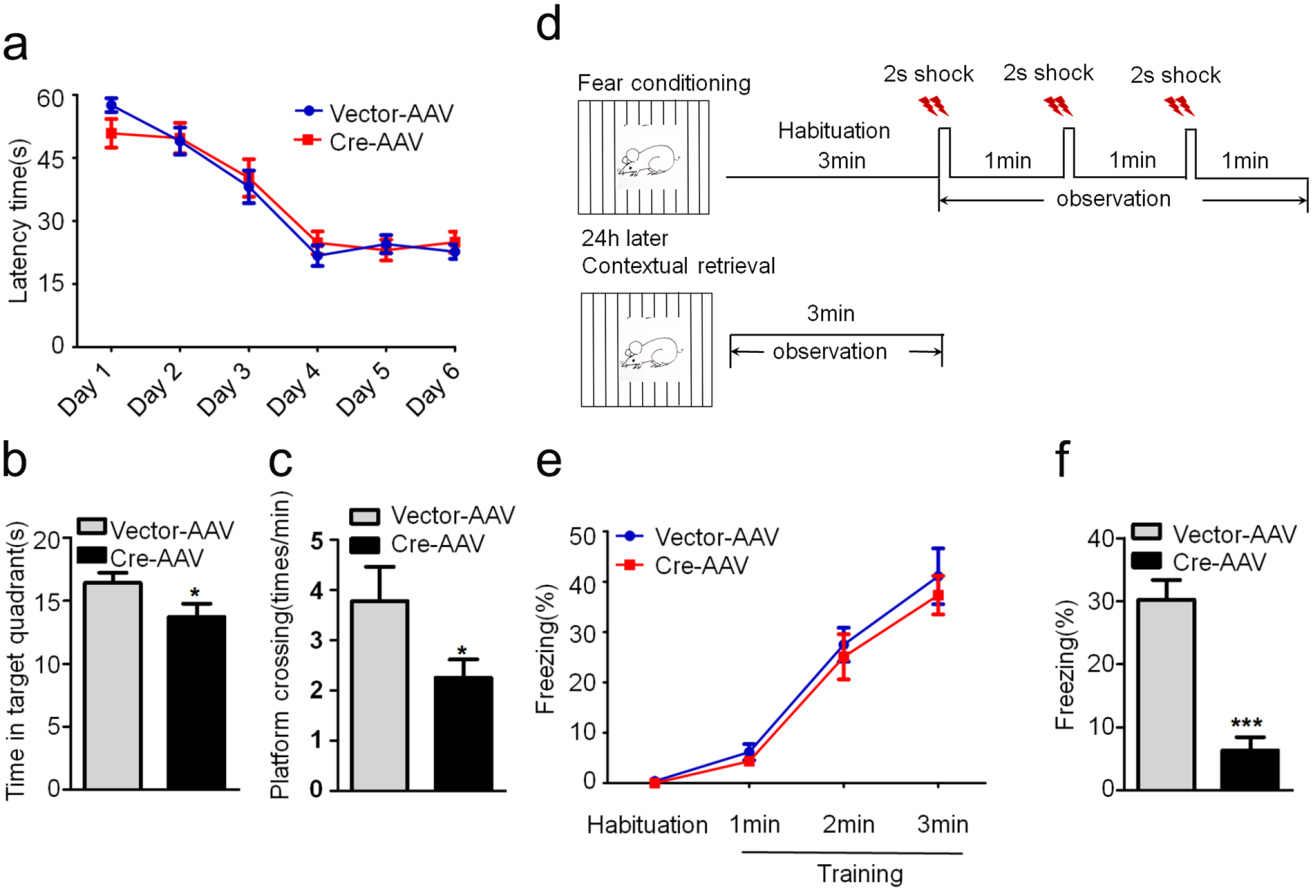
GSK-3β deletion in DG induces spatial and fear memory deficits. (**a**) GSK-3β deletion in DG excitatory neurons does not change the latency to find the hidden platform during 6 days Morris water maze (MWM) training. (**b,c**) GSK-3β deletion induces memory deficits shown by the decreased time in previous target quadrant and the crossings in the platform region measured at day 8 by removed the platform (Vector, n = 10; Cre, n = 8). (**d**) The schematic representation of contextual fear memory test. (**e**) No change of freezing time was detected in GSK-3β deletion during the contextual fear training test. (**f**) The percentage time of freezing was remarkably decreased during the contextual memory test carried out in the next day (Vector, n = 10; Cre, n = 8). Data were presented as mean ± s.e.m. Two–way repeated-measures ANOVA with Huynh-Feldt-Lecoutre correction for panels (a) and (e); unpaired t test for panels (b, c, and f). *P < 0.05, ***P < 0.001 *versus* Vector. The absence of asterix indicates that the difference is not significant.

**Figure 3:**
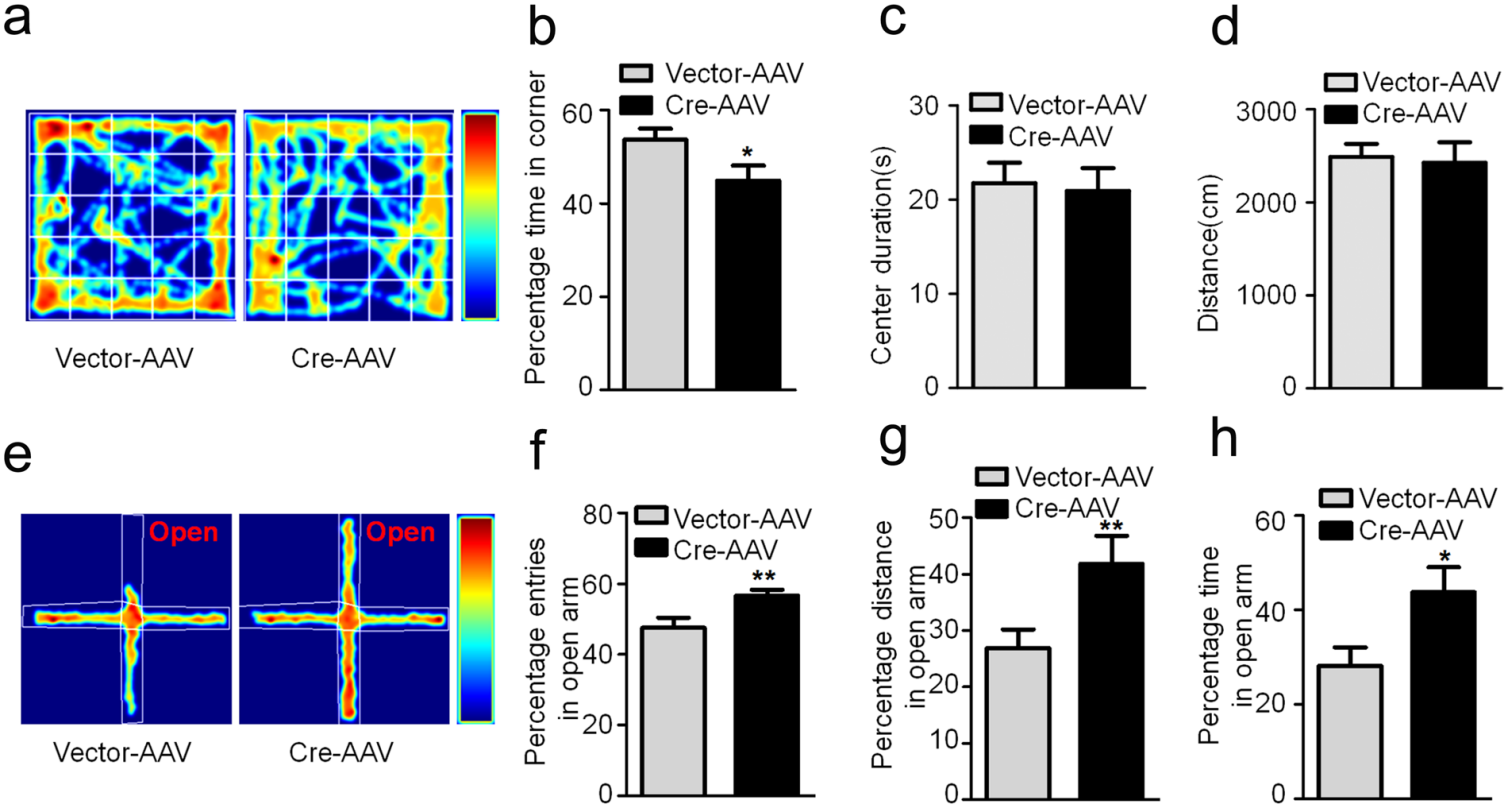
GSK-3β deletion in DG induces an anti-anxiety behavior. (**a**) The representative route recorded in open field test. (**b–d**) GSK-3β deletion reduced the percentage time spent in corner without changing the time spent in the center and the total distance moved in the open field test.(Vector, n = 10; Cre, n = 8, unpaired t test) (**e**) The representative route in the elevated maze test. (**f–h**) GSK-3β deletion increased the percentage of open arm entries, the percentage distance and total time spent in the open arm(Vector, n = 10; Cre, n = 8, unpaired t test). Data were presented as mean ± s.e.m. *P < 0.05, **P < 0.01 *versus* Vector; the absence of asterix indicates that the difference is not significant.

The hippocampal DG subset is critical in spatial and fear memory^30, 35, 36^. Therefore, we first measured spatial memory of the mice by MWM test. During the first 6 days training sections, we did not observe significant difference in latency to find the submerged platform between two groups (Fig. 2a). However, the mice with GSK-3β deletion showed decreased time spent in the target quadrant and a decreased platform crossing in the probe trial during spatial memory test (Fig. 2b,c). In contextual fear memory test, GSK-3β deletion did not change freezing response during training test, but the freezing time was remarkably decreased during contextual memory test measured at 24 h after (Fig. 2e,f). These data indicate that GSK-3β deletion in DG excitatory neurons impairs spatial and contextual memory without significantly changing learning ability of the mice. As hippocampal DG subset and GSK-3β are closely involved in anxiety^37–39^, we also measured the anxiety behavior. The results showed that GSK-3β deletion in DG excitatory neurons significantly decreased corner duration in open field test (Fig. 3a–d), and increased the staying time in open arm during elevated maze test (Fig. 3e–h). These data suggest that GSK-3β deletion can decrease anxiety level.

### GSK-3β deletion decreases synapse proteins and impairs synaptic plasticity

**Figure 4:**
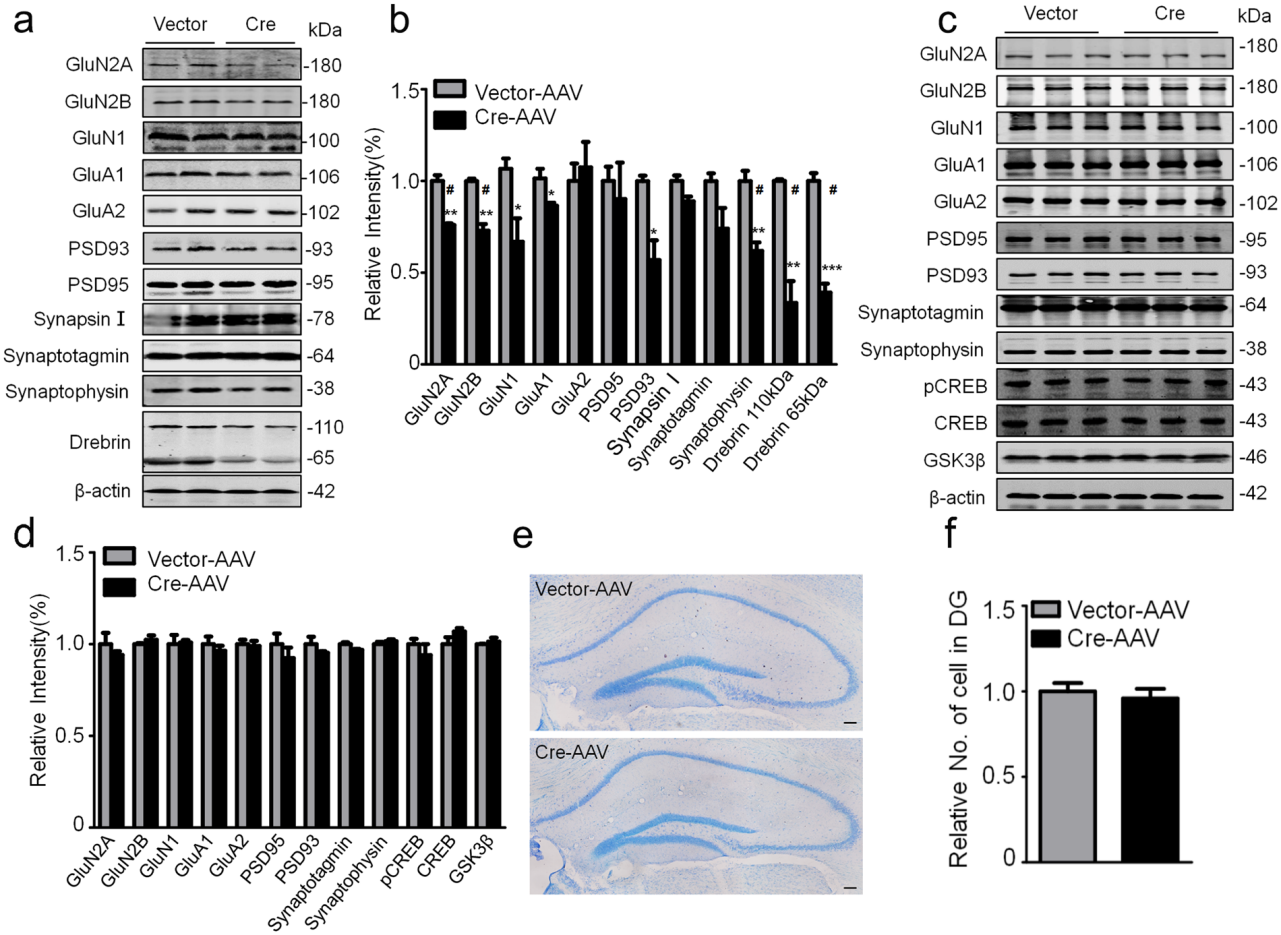
GSK-3β deletion in DG decreases several synapse-associated proteins. (**a,b**) GSK-3β deletion decreased the levels of multiple pre- and post-synaptic proteins in DG extracts measured by Western blotting and the quantitative analyses (n = 4 each group) (* by unpaired t test, # by multiple t-tests using Sidak-Bonferroin method of GraphPad Prism 6.0). (**c,d**) GSK-3β deletion in DG excitatory neurons did not change the synapse-associated protein in prefrontal cortex (n = 3 each group) (* by unpaired t test, ^#^ by multiple t-tests using Sidak-Bonferroin method of GraphPad Prism 6.0). (**e,f**) The representative images of the hippocampus by Nissl staining and the densitometric analyses (n = 5–6 each group, unpaired t test). Scale bars, 100 μm. Data were presented as mean ± s.e.m. *P < 0.05, **P < 0.01,***P < 0.001 *versus* Vector. “#” indicates that the difference is significant analyzed by multiple t-tests. The absence of asterix indicates that the difference is not significant.

Synaptic plasticity, including expression of synaptic related proteins, spine density and morphology, and the functional neuronal transmission, is the precondition of cognitive abilities^40, 41^. To explore the mechanisms that may underlie the behavioral impairments induced by GSK-3β deletion, we measured the levels of synaptic proteins. By unpaired t-test analysis, we observed that GSK-3β deletion remarkably decreased the levels of GluN1, GluN2A and GluN2B (NMDAR subunits), GluA1 (AMPAR subunit), PSD93 and drebrin (postsynaptic structural proteins), and synaptophysin (presynaptic protein) in DG subset (Fig. 4a,b), while the levels of these proteins were not changed in the prefrontal cortex (Fig. 4c,d). By using multiple comparisons, the difference of GluN2A, GluN2B, drebrin and synaptophysin reductions was still significant (Fig. 4a,b). No significant neuron loss was shown upon GSK-3β deletion in DG subset measured by Nissl staining and densitometric analyses (Fig. 4e,f).

**Figure 5:**
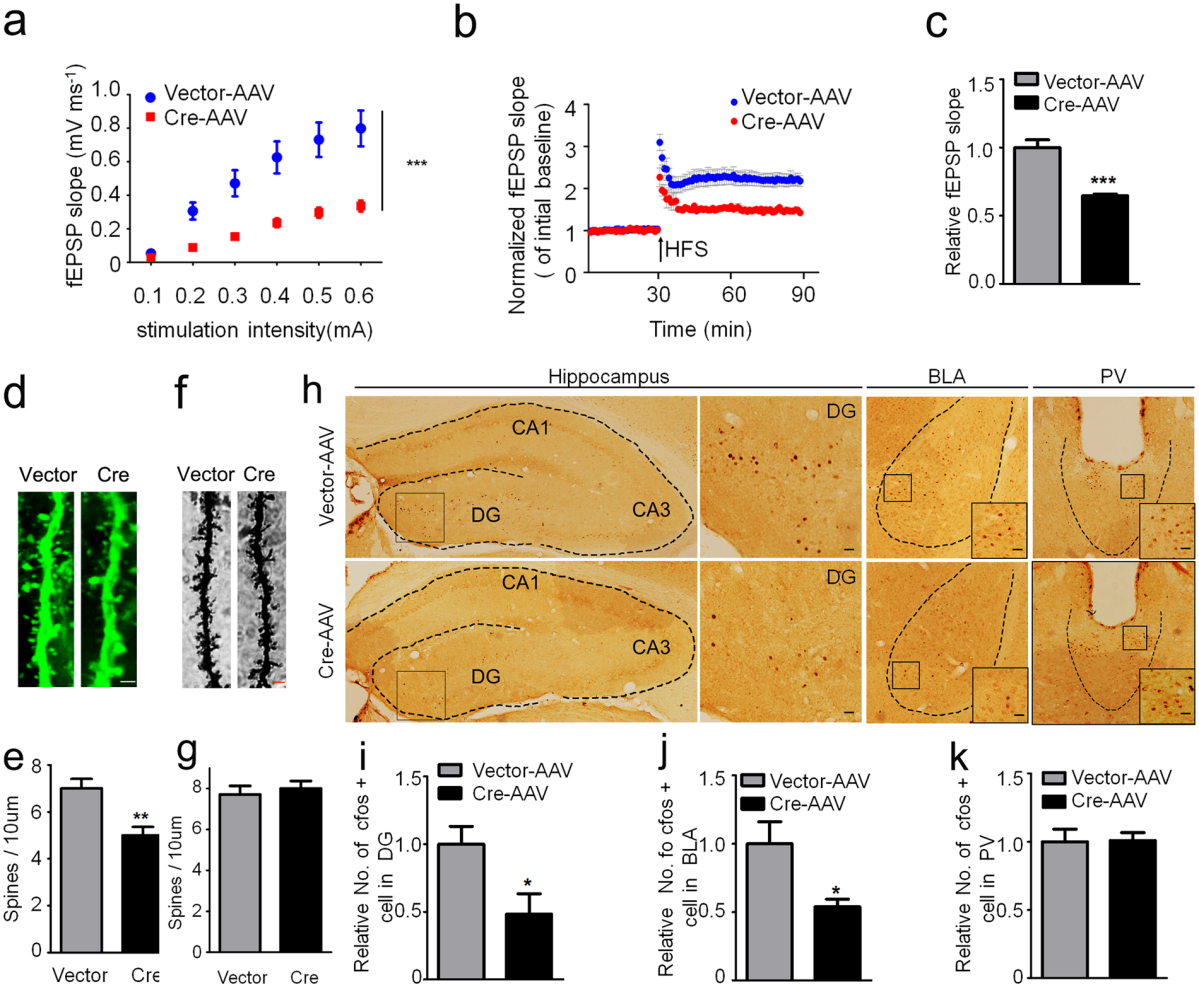
GSK-3β deletion inhibits activity-dependent neural activation. (**a**) GSK-3β deletion significantly suppressed basal synaptic transmission shown by the reduced input/output (I/O) curve. (**b,c**) GSK-3β deletion induced LTP impairment shown by a decreased slop of the evoked fEPSP, and the decrease was still significant at 60 min after high frequency stimulation (HFS). n = 8–10 hippocampal slices from 6 mice in each group. (**d,e**) The representative micrographs of spine morphology and the decreased spine density in GFP-positive neurons (6 mice were analyzed in each group). (**f,g**) The representative micrographs of spine morphology and the spine density in prefrontal cortex neurons (6 mice were analyzed in each group). (**h–k**) The representative immunohistochemical images of c-fos, and the densitometric analyses in DG (dentate gyrus), BLA (basal lateral amygdala) and PV (paraventricular thalamic nucleus) subsets (Scale bars, 20 μm; n = 5–6 each group). Data were presented as mean ± s.e.m. Two–way repeated-measures ANOVA with Huynh-Feldt-Lecoutre correction for panels (**a**), unpaired t test for panels (**c**,**e**,**i**–**k**). *P < 0.05, **P < 0.01. ***P < 0.001 *versus* Vector. The absence of asterix indicates that the difference is not significant.

By electrophysiological recording on acute brain slices, the basal synaptic transmission and LTP induction from DG to CA3 subset were inhibited upon GSK-3β deletion (Fig. 5a–c). GSK-3β deletion also decreased the number of dendritic spine in DG excitatory neurons measured by z-stack scanning under a two-photon fluorescence microscope (Fig. 5d,e), while the GSK-3β deletion in DG did not affect the spine density in prefrontal cortex (Fig. 5f,g). To further explore the mechanisms that may underlie the synapse impairments, we measured neural activity by c-fos staining. The number of c-fos-positive neurons in DG and basal lateral amygdala (BLA) subsets was significantly decreased (Fig. 5h–j). As a negative control, no difference of c-fos-positive neurons was observed in subset of paraventricular thalamic nucleus (PV) in GSK-3β deletion group (Fig. 5h,k). These data together suggest that GSK-3β deletion in DG excitatory neurons impairs synaptic plasticity and arrests the activity-dependent neural activation in DG and BLA.

### GSK-3β deletion inhibits CaMKII/IV-CREB signaling

**Figure 6:**
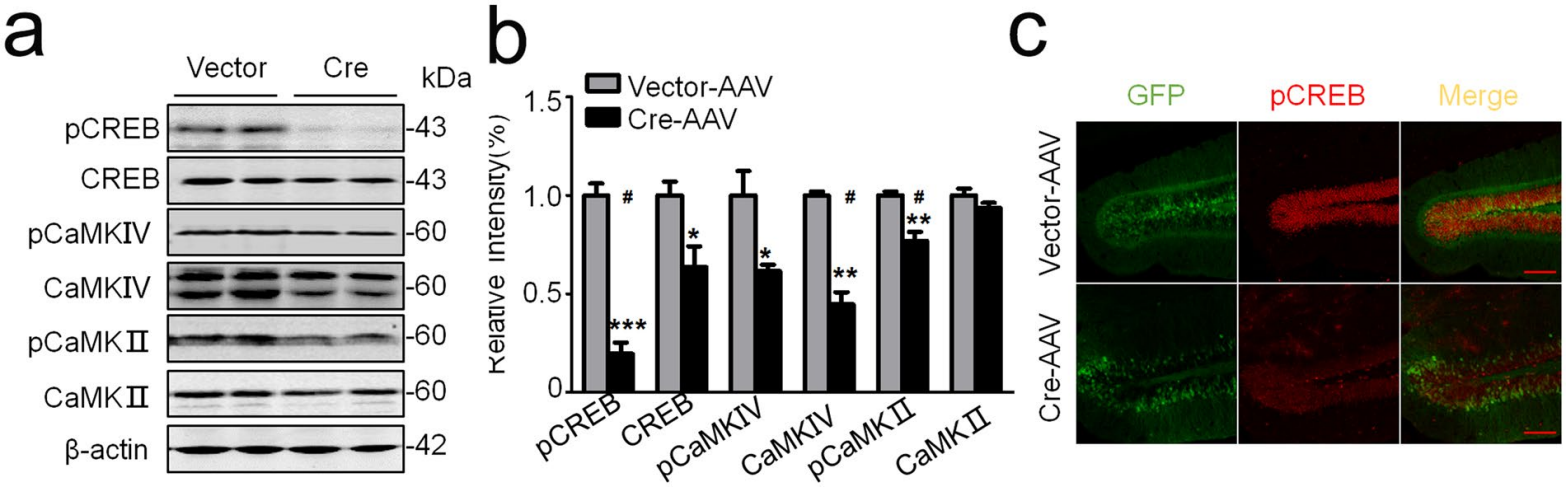
GSK-3β deletion inhibits CaMKII/IV-CREB signaling. (**a,b**) GSK-3β deletion decreased total and/or the phosphorylated levels of CaMKII, CaMKIV and CREB in DG extracts measured by Western blotting and the quantitative analyses. (**c**) The reduced intensity of pCREB in DG excitatory neurons was also shown by co-immunofluorescent staining. Scale bars, 100 μm. Data were presented as mean ± s.e.m. n = 4 each group, analyzed by unpaired t-test (*) or multiple t-tests (#). *P < 0.05, **P < 0.01, *** < 0.001 *versus* Vector, “#” indicates that the difference is significant analyzed by multiple t-tests. the absence of asterix indicates that the difference is not significant.

The CaMKII/IV-CREB signaling plays a crucial role in synapse-associated protein expression^42, 43^. Therefore, we also measured the effects of GSK-3β deletion on CaMKII/IV-CREB signaling. We observed that the total protein level and/or the phosphorylated levels of CREB, CaMKII and CaMKIV were all decreased in GSK-3β deleted DG subset of the hippocampus and reduction of the phosphorylation (the active forms) was more significant, in which pCREB was almost completely diminished (Fig. 6a,b). Significant reduction of pCREB in hippocampal DG was also detected by immunofluorescence staining in GSK-3β deletion group (Fig. 6c). These data suggest that GSK-3β deletion in DG may inhibit expression of synaptic proteins with CaMKII/IV-CREB-associated mechanisms.

## Discussion

As GSK-3β knockout in mouse is lethal during the embryonic period^23^, pharmacological inhibitors^44–46^ and shRNA-mediated silencing^33^ have been widely used to downregulate the kinase in previous studies. The transgenic approaches have also been employed to knockdown GSK-3β in pan-neurons^24, 25^. By using Cre-loxp system, this is the first report showing that selective deletion of GSK-3β in hippocampal DG excitatory neurons induces spatial and fear memory deficits, and the mechanisms involves inhibition of CaMKII/CaMKIV/CREB signaling-related neural activation and synaptic plasticity.

GSK-3β is abundantly expressed in hippocampus^47^. In hippocampal formation, CA3 subset plays important role in encoding new spatial information, which requires multiple trial learning and short-term memory retrieval based on a spatial pattern completion process, while the CA1 subset receives incoming information from CA3 and sets up associatively learned back projections to entorhinal cortex that is consistent on consolidation^48^. We choose to delete GSK-3β in DG because it is a critical subset for spatial and fear memory and for psychiatric behaviors^36, 49–51^. In MWM test, we observed no significant difference between groups in the training section, but the mice with GSK-3β deletion displayed memory impairment in the probe trials. In contextual fear conditioning test, GSK-3β deletion did not affect the foot shock response during training, but severely impaired the fear memory. In a previous report, DG pan-neuronal GSK-3β knockdown by lentivirus did not alter the memory in MWM test^33^. We speculate that the excitatory neuron-specific deletion of GSK-3β *versus* pan-neuronal manipulation may explain the discrepancy. Additionally, the AAV used in the current paper achieved ~74% knockdown in the excitatory neurons, while the lentivirus employed in the previous paper only achieved ~20% knockdown of GSK-3β in pan-neurons.

Neuronal excitation and the Ca^2+^ signaling pathway are the bases of neural transmission and the activity-dependent protein synthesis^42, 43, 52^. Previous studies demonstrate that GSK-3β is critical for rhythms-related neuronal activity^22^. GSK-3β affects endocytosis and exocytosis^5, 53^. Hence, we hypothesize that GSK-3β knockout may alter neuronal activity in response to the cognitive manipulations. Our results did show that neuron activation during fear memory was mainly located in DG, however, the number of c-fos-positive neurons was markedly decreased by GSK-3β deletion. This result indicates that GSK-3β deletion alters the neuronal activity in DG during learning and memory. GSK-3β deletion did not induce significant cell loss measured by Nissl staining. Nonetheless, we do not exclude the possibility that the compromised neuronal survival by GSK-3β deletion could be compensated by an increased neurogenesis in the region. Indeed, inhibition of GSK-3 has been shown to stimulate adult neurogenesis^54^. Therefore, a higher turnover of DG granule cells in GSK-3β conditional deletion may be present, which deserves further investigation.

We also observed that the neuronal activity in BLA, a region related with fear memory^55^, significant decreased in mice with GSK-3β deletion in DG excitatory neurons. Currently, it is not known whether BLA receives axonal projections from DG. However, as hippocampal trisynaptic circuit (DG-CA3-CA1) involves CA1^29^ and BLA receives axonal projection form CA1^56^, by which DG and BLA subsets may have indirect connection, and GSK-3β deletion in DG excitatory neurons may impair this connection.

CaMKII/IV is related with the complexity of neuronal dendritic spines^57, 58^. We observed that selective deletion of GSK-3β in DG excitatory neurons inhibited CaMKII activity with reduction of total and the phosphorylated CaMKIV. It is currently not understood how GSK-3β deletion leads to suppression of CaMKII/IV. As CREB is robustly phosphorylated by CaMKII and CaMKIV, which regulates the transcription of neuronal activity-related proteins^42, 43^, we speculate that cross-talk of CAMKII/IV and CREB inhibition may at least contribute to the significantly reduced expression levels of multiple synaptic proteins. We also noticed that GSK-3β deletion did not significantly change the expression levels of GluA2 and PSD95, suggesting that GSK-3β may regulate different synapse-associated proteins with distinct mechanisms, which may be investigated in future studies.

Previous studies demonstrate that GSK-3β activation inhibits LTP induction^6^, while pharmacological inhibition and lentivirus silencing of GSK-3β potentiates LTP^33, 59^. Here, we observed that selective deletion of GSK-3β impaired dendrite density in DG excitatory neurons with inhibition of LTP induction from DG to CA3, suggesting that GSK-3β in DG excitatory neurons plays a critical role in dendrite development and presynaptic transmission of DG-CA3. With regard to GSK-3β on LTP, it was also reported previously that pharmacological blockade of the hyperactivated GSK-3β by lithium or SB216763 or transient expression of a dominant-negative GSK-3β mutant attenuated LTP deficits of from medial perforant path (MPP) input to DG or MPP-CA3 or Schaffer collateral (SC) synapses in CA1 pathways in Fragile-X mice^59^ or in rats^6^ or mice^60^. On the other hand, Chew *et al*. reported that knockdown of GSK-3β by expressing lenti-ShRNA in DG pan-neuronal cells enhanced postsynaptic LTP from MPP input to DG^33^. We speculate that the following differences may at least partially explain the discrepancy: (i) non-specific pan-neuronal partial knockdown (~20%) of GSK-3β in previous study *versus* specific excitatory neuronal deletion in current paper. (ii) the postsynaptic LTP of MPP-DG or MPP-CA3 measured by previous study *versus* presynaptic LTP of DG-CA3 measured in current paper, which may represent different synaptic plasticity^61^. A most recent study shows that GSK-3β deletion impairs the amplitude and frequency of AMPAR-dependent synaptic currents^25^, which supports our current finding. Nonetheless, the pharmacological approaches and shRNA experiments could be more acute than conditional deletion, while the latter may induce risk of measuring secondary effects of GSK-3β deletion.

Taken together, we have observed that selective deletion of GSK-3β in DG excitatory neurons induces memory deficit and anxiety-like behavior with mechanisms involving impaired synaptic plasticity and neural inactivation.

## Materials and Methods

### Animals, viruses, and stereotaxic surgery

The GSK-3β floxed mouse (GSK-3β^lox/lox^) is a kind gift of Dr. James R Woodgett^34^. The mice were housed under a 12-h light/dark cycle and kept with accessible food and water at 25 °C. All animal studies were approved by the Ethics Committee of Tongji Medical College, Huazhong University of Science and Technology. The AAV-CaMKII-Cre-2A-eGFP vector was designed for the specific expression of Cre recombinase with non-fusion eGFP in excitatory neurons. For brain injection, the mice (2m-old) were fixed in the stereotaxic apparatus (DAVID kopf instruments) and anesthetized with Isoflurane (2.0% for induction and 1.5% for maintenance). After being sterilized with iodophors, the scalp was incised along skull midline and holes were drilled in the hibateral stereotaxically at posterior 1.9 mm, lateral 1.1 mm, and ventral 2.0 mm relative to bregma. Using an automatic microinjection system (World Precision Instruments), AAV-CaMKII-Cre-2A-eGFP or the empty vector (1 μl, 4.0 × 10^12^ viral particles per ml) was injected into the hippocampus DG region at a rate of 0.1 μl/min with Hamilton needle, the needle was kept in place for 10 min before slowly pull out, the skin was sutured, and sterilized with iodophors, and the mice were placed in thermo tank for analepsia. At 4 weeks after brain infusion of the virus, the behaviors were measured as described as follows.

### Morris water maze (MWM) test

The MWM test was performed as described in a previous study^62, 63^. The mice were trained to find a hidden platform which submerged under milk water for six consecutive days, three trials per day, and cues outside the pool were constant. During each trial, the mouse started from one of three quadrants (exclude target quadrant) facing the wall of the pool and find the hidden platform in 60 s, after which they were guided to the platform and placed for 20 s. The swimming path and latency were recorded in each trail by a digital video camera connected to a computer (Chengdu Taimeng Software Co. Lid, China). On the 8th day, the mice were allowed to swim for 60 s without the hidden platform. The time stayed in the previous target quadrant and the number of the target platform crossings was recorded.

### Contextual fear conditioning

The mice were placed into the conditioning chamber and allowed to explore the conditioning chamber for 3 min. Then they received three times electric foot shocks (0.8 mA, 2 s each, and 1 min rest in between), and then they were returned to home cages. The chambers were cleaned with 75% alcohol before the next mouse was trained. After 24 h, the mice were placed back into the conditioning chamber for 3 min without electric foot shock. The activity and freezing behavior were recorded by a video tracking system (Chengdu Taimeng Software Co. Lid, China).

### Open field test

The Open-Field test consisted of a 5-min session in one 50 × 50 cm chamber (white opaque plastic), which was divided into 25 square regions, a central field (center 9 square regions, 15 × 15 cm^2^) and a periphery field. Each mouse was placed in the same periphery region at the start of the test. Behaviors were recorded and analyzed by the video tracking system (Chengdu Taimeng Software Co. Lid, China).

### Elevated plus maze

The Elevated plus maze test was performed as described in a previous study^64^. Briefly, mice were placed at the junction of the four arms of the maze (white opaque plastic), and the starting test was faced an open arm. The chambers were cleaned with 75% alcohol before the next mouse was trained. And the entries and/or duration in each arm were recorded for 5 min by the video tracking system (Chengdu Taimeng Software Co. Lid, China). An increase in open arm activity (duration and/or entries) reflects anti-anxiety behavior.

For animal studies, the mice (all had the same gene background) were randomly divided into experimental and the control groups and the behavioral tests were carried by the same operators who were blind of the treatments the mice received. After behavior tests, the virus expression and the location were confirmed by fluorescence microscopy. The mice lacking robust virus expression or having virus spreading onto the other brain regions were discarded from behavioral data analysis (~20%).

### Western blotting

The western blotting was performed as described in a previous study^62^. The mice were anesthetized with an i.p. injection of 100 mg/kg of ketamine and 50 mg/kg of xylazine. The mice brains sectioned (300-μm-thick) using vibrating microtome (Leica, VT1000 S, Germany) on ice-cold PBS, and the hippocampus DG subset was accurately separated according to the mouse brain atlas. The proteins of hippocampus DG were extracted using RIPA buffer (P0013B, Beyotime) and the concentration was determined by BCA method. Equal amount of proteins were separated by 10% SDS–polyacrylamide gel electrophoresis (SDS-PAGE) and transferred onto the nitrocellulose membranes, blocked with 5% skim milk for 1 h at room temperature, and then incubated with primary antibodies (Table 1) for 2 h at room temperature or overnight at 4 °C. The bands were visualized by using Odyssey Infrared Imaging System (LI-COR biosciences, Lincoln, NE, USA). The protein of hippocampal extracts loaded for Western blotting was 15 μg for GSK-3β and GSK-3α; 10 μg for synapsin I, synaptotagmin, synaptophysin; 5 μg for β-actin; 30 μg for GluN1; and 20 μg for other proteins. For cortex analysis, 30 μg for GluN2A, GluN2B, GluA1 and GluA2; and 20 μg for all other proteins.

**Table 1:** Antibodies employed in the study.

Antibody	Specificity/Immunogen	Host	Dilution	Catalogue numbers
GluN1	Total/human aa 850–950	R	1:500 WB	Abcam ab109182
GluN2A	Total/mouse C terminal last 200 aa	R	1:1000 WB	Abcam ab14596
GluN2B	Total/rat aa 1450 to C-terminu	R	1:1000 WB	Abcam ab65783
GluA1	Total/human aa 840–850	R	1:1000 WB	Millpore 04–855
GluA2	Total/rat C-terminus	R	1:1000 WB	Millpore AB10529
PSD95	Total/human PSD95	R	1:1000 WB	Cell signaling 2507
PSD93	Total/human aa surrounding pro321	R	1:1000 WB	Cell signaling 19046
Synapsin I	Total/mixture of Ia & Ib of bovine brain	R	1:1000 WB	Millpore AB1543
Synaptotagmin	Total/mouse aa 100–200	R	1:1000 WB	Abcam ab106621
Synaptophysin	Total/human aa 250–350	R	1:1000 WB	Abcam ab32127
Drebrin	Total/Chicken Drebrin	M	1:1000 WB	Abcam ab12350
pCREB	Phosphorylated at Ser133/human	R	1:1000 WB	ThermoFisher MA5-11192
			1:200 IF	
CREB	Total/human CREB	R	1:1000 WB	Abcam ab31387
pCaMKIV	Phosphorylated at Thr196/human	R	1:1000 WB	Santa cruz sc-28443
CaMKIV	Total/aa 1–241	M	1:1000 WB	Santa cruz sc-136249
pCaMKII	Phosphorylated at Thr286/human	R	1:500 WB	Cell signaling 3361
CaMKII	Total/human	R	1:500 WB	Cell signaling 3362
CaMKII	Total/mouse (6G9)	M	1:100 IF	Cell signaling 50049
β-actin	Total/aa 1–14	M	1:1000 WB	Abcam ab6276
C-fos	Total/human N terminal	R	1:50 IH	Santa cruz sc-52
GSK-3β	Total/human C-terminus (D5C5Z)	R	1:1000 WB	Cell signaling 12456 (Figs 1f, 4c, Supplementary Fig. 2)
			1:100 IH	
GSK-3β	Total/human C-terminus	G	1:500 WB	Santa cruz sc-8257 (Fig. 1c)
GSK-3α	Total/human C-terminus (D80E6)	R	1:1000 WB	Cell signaling 4337
GAD67	Total/ human aa 87–106	M	1:50 IF	Abcam ab26116

WB: Western blotting; IH: immunohistochemistry; IF: Immunofluorescence; aa: amino acid; M: mouse; R: rabbit; G: goat.

### Immunohistochemistry and immunofluorescence

The mice were anesthetized with an i.p. injection of 100 mg/kg of ketamine and 50 mg/kg of xylazine, and transcardially perfused with normal saline (NS), followed by 4% paraformaldehyde (PFA) for 30 min. Brains were removed and post-fixed for additional 48 h. After that, the brains were sliced 30 μm with a vibrating microtome (VT1000S, Leica, Germany). The brain slices permeabilized in phosphate buffer containing 0.5% Triton X-100, 3% H_2_O_2_ for 30 min, then blocked by 3% BSA for 30 min. The slices were incubated with primary antibody shown in Table 1 for overnight at 4 °C, followed by a horseradish peroxidase-labeled antibody for 1 h at 37 °C, and exposed to DAB. The images were observed under a microscope (Nikon, 90i, Tokyo, Japan). The c-fos staining was analyzed in BLA (Coronal section, −1.4 mm Bregma). For immunofluorescence, the brain slices were incubated with donkey anti-Rabbit IgG (H + L) secondary antibody alexa Fluor 546 (ThermoFisher Scientific, A10040) for 1 h at room temperature, and the images were observed with a laser confocal microscope (710; Zeiss, Germany).

### Nissl staining

The mice were anesthetized with an i.p. injection of 100 mg/kg of ketamine and 50 mg/kg of xylazine, and were fixed by perfusing 4% paraformaldehyde solution through heart. The brain was removed and post-fixed in the same fixative solution for overnight at 4 °C. Coronal sections (30 μm thickness) were prepared by using vibratome. The sections were submerged in 0.5% cresyl violet solution for about 1 min.

### Electrophysiology

Mice were deeply anesthetized with an i.p. injection of 100 mg/kg of ketamine and 50 mg/kg of xylazine, and the brains were immediately removed and immersed in ice-cold oxygenated artificial cerebrospinal fluid (ACSF; 2.0 mM KCl,125 mM NaCl, 1.2 mM MgSO_4_, 26 mM NaHCO_3_, 1.2 mM KH_2_PO_4_, 2.5 mM CaCl_2_ and 11 mM glucose). Parasagittal sections (300 μm) were cut using a vibrating microtome (Leica VT1000S, Leica Biosystems) at 4–5 °C in ACSF and the slices were pre-incubated in oxygenated ACSF at 30 °C for at least 60 min. Then one slice was laid down in the recording chamber (an 8 × 8 microelectrode array) submerged in ACSF (1 ml min^−1^) with temperature at 34 °C. MED64 system (Alpha MED Sciences, Panasonic) was used to record the fEPSPs in CA3 neurons by stimulating the mossy fibers from DG. LTP was induced by applying three trains of high-frequency stimulation (HFS; 100 Hz, 1-s duration), stimulation strength was set to 40% of the maximum obtained by plotting an input–output curve.

### Spine analyses

Golgi staining for prefrontal cortex: The mice were deeply anesthetized and then fixed by transcardial perfusion with 0.5% NaNO_2_ followed by 4% formaldehyde and potassium dichromate with chloral hydrate which were mixed in 4% formaldehyde. After perfusion, the brains were postfixed in potassium dichromate with chloral hydrate mixed liquid for 3 days. Then the brains were moved into 1% AgNO3 solution for 3 days. Coronal sections of the brain were cut (100 μm thick) using Vibratome microtome (Leica, VT1000 S, Germany).

GFP-spine acquisition and analysis: The mice were anesthetized with an i.p. injection of 100 mg/kg of ketamine and 50 mg/kg of xylazine, and transcardially perfused with normal saline (NS), followed by 4% paraformaldehyde (PFA) for 30 min. Brains were removed and post-fixed for additional 48 h. After that, the brains were sliced 30 μm with a vibrating microtome (VT1000S, Leica, Germany). The slices were attached to slide glass, covered with 30% glycerol and coverslips. The spine image acquisition and analysis were performed as described in a previous study^25^. The excitatory neurons expressing non-fused GFP were used for spine counting. A Zeiss 100× immersion objective (Zeiss LSM710, Carl Zeiss AG, Oberkochen, Germany) was used to acquire images with 0.5 µm z-resolution.

Both Golgi staining and GFP-positive neuronal spine densities were determined in segments of dendrites at a distance of 90 μm from the soma, counted in z-stacks by manual scrolling of the images. Spine densities refer to the amount of spines per 10 μm dendrite length analyzed by using Imaris software (Bitplane, Zürich, Switzerland).

### GSK-3β activity assay

The activity of GSK-3β was assayed using a kit (GMS50161.6, Genmed) by following the manufacturer’s instructions.

### Statistics

Data were expressed as mean ± s.e.m. and analyzed using SPSS version 21.0 for Windows (SPSS Inc., Chicago, IL, USA) for The two–way repeated-measures ANOVA or Student’s t–test and GraphPad Prism 6.0 (GraphPad Software, Inc, La Jolla, CA) for Multiple t-test. The level of significance was set at *p* < 0.05.

### Availability of data and material

The authors declare that the data supporting the findings of this study are available within the article and its Supplementary Information files, or from the authors upon request.

### Ethics approval and consent to participate

All animal experiments were performed according to the ‘Policies on the Use of Animals and Humans in Neuroscience Research’ revised and approved by the Society for Neuroscience in 1995, and the animal study was approved by the Academic Review Board of Tongji Medical College, Huazhong University of Science and Technology.

## Electronic supplementary material

Supplementary Information
