# Supplementary material for: GSK-3β deletion in dentate gyrus excitatory neuron impairs synaptic plasticity and memory
Source: Sci Rep. 2017 Jul 18;7:5781. doi: 10.1038/s41598-017-06173-4 (PMC5515925; doi:10.1038/s41598-017-06173-4)

GSK-3β deletion in dentate gyrus excitatory neuron impairs synaptic plasticity and memory

Enjie Liu**^1^,***, Ao-Ji Xie^1^**,***, Qiuzhi Zhou**^1^**, Mengzhu Li**^1^**, Shujuan Zhang**^1^**, Shihong Li**^1^**, Weijin Wang**^1^**, Xiaochuan Wang**^1^**, Qun Wang**^1^**, Jian-Zhi Wang**^1,2^**

**^1^**Department of Pathophysiology, School of Basic Medicine and the Collaborative Innovation Center for Brain Science, Key Laboratory of Ministry of Education of China for Neurological Disorders, Tongji Medical College, Huazhong University of Science and Technology, Wuhan, 430030, PR China.

**^2^** Co-innovation Center of Neuroregeneration, Nantong, 226000, PR China.

*Equally contributed to the paper.

Correspondence should be addressed to J.Z.W. ([wangjz@mails.tjmu.edu.cn](mailto:wangjz@mails.tjmu.edu.cn)).

**Supplementary Figure 1**

**
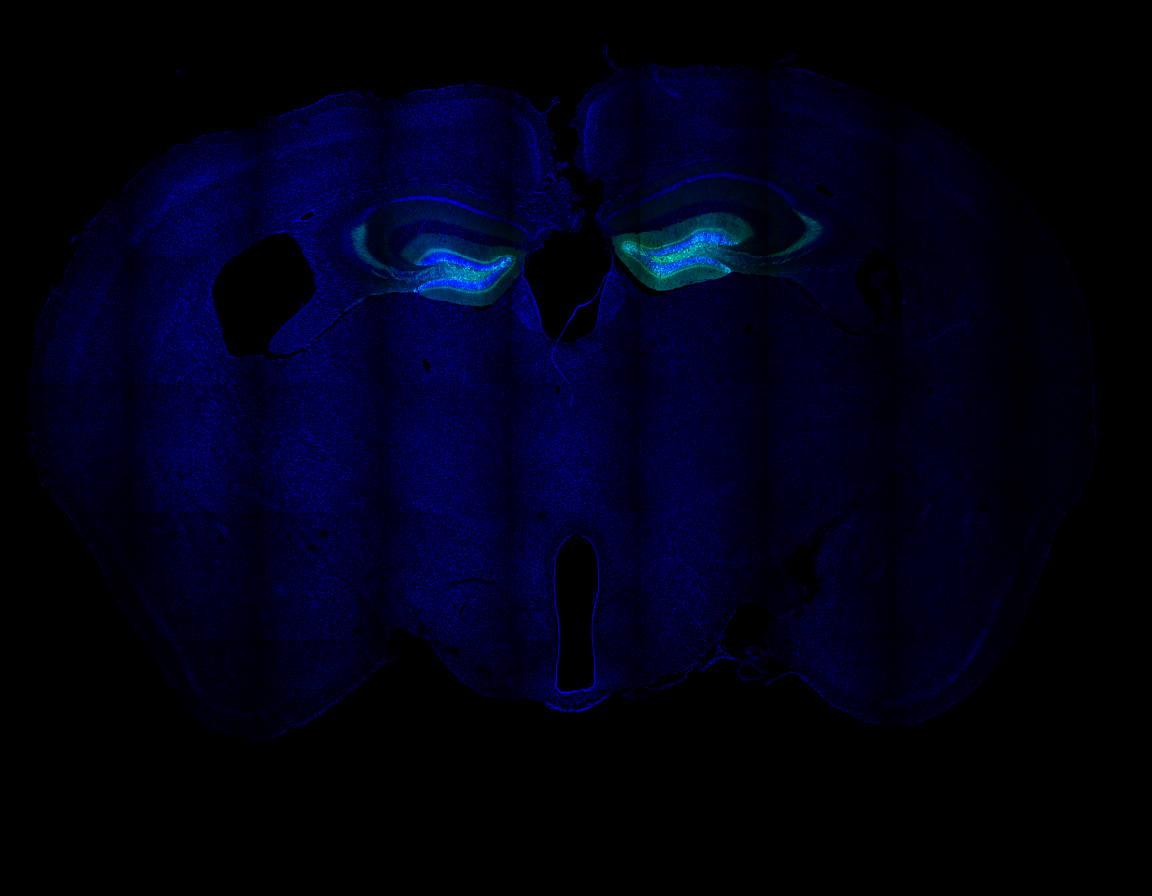
**

**Supplementary Figure 1.** The representative imaging showing full region of virus injections, the blue is hoechest stained, the green indicates viral infection area.

**Supplementary Figure 2
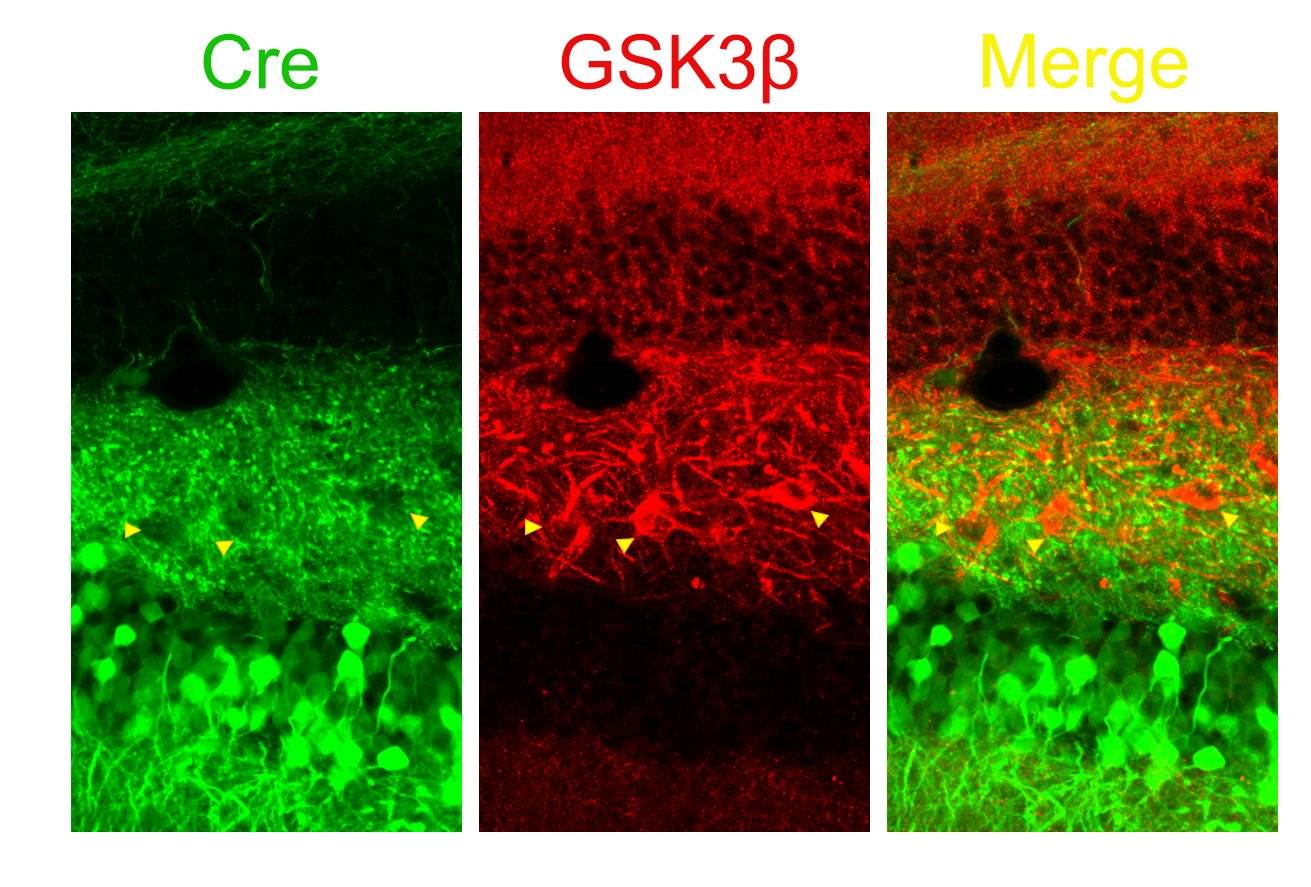
**

**Supplementary Figure 2.** The representative immunofluorescence staining showing selective deletion of GSK-3β in neurons infected with AAV-CaMKII-Cre. AAV-CaMKII-Cre (0.4 μl) was injected/expressed into part of the hippocampal excitatory neurons in dentate gyrus (Cre). The immunofluorescent data showed that GSK-3β was selectively deleted in the virus-infected neurons but not in the other uninfected regions (marked with a triangular). Note that the co-localization of red and green maybe due to the overlapping signal, and the minor GSK-3β staining in the virus-infected region maybe due to the projection of other neurons.

**Supplementary Figure 3. Full-length blots for Figure 1 c, i.**


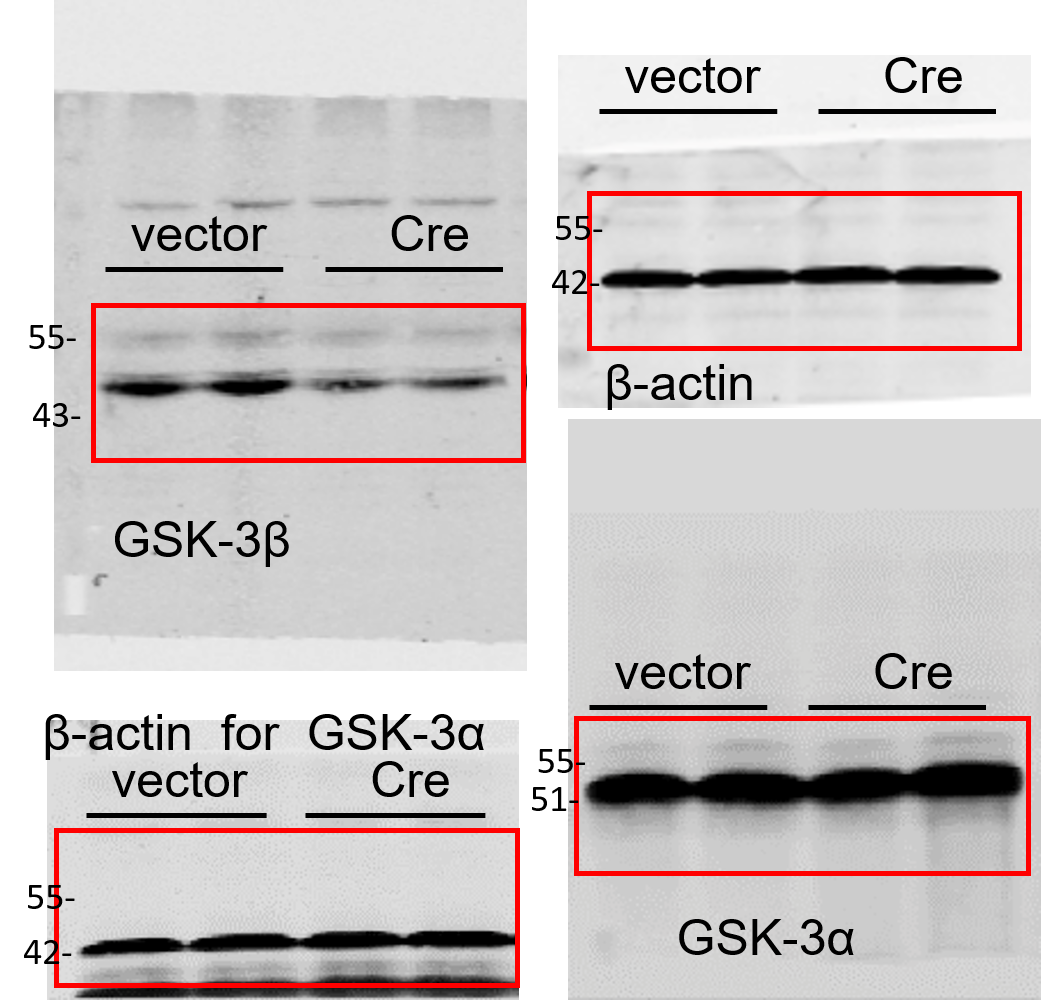


**Supplementary Figure 4. Full-length blots for Figure 4a.**


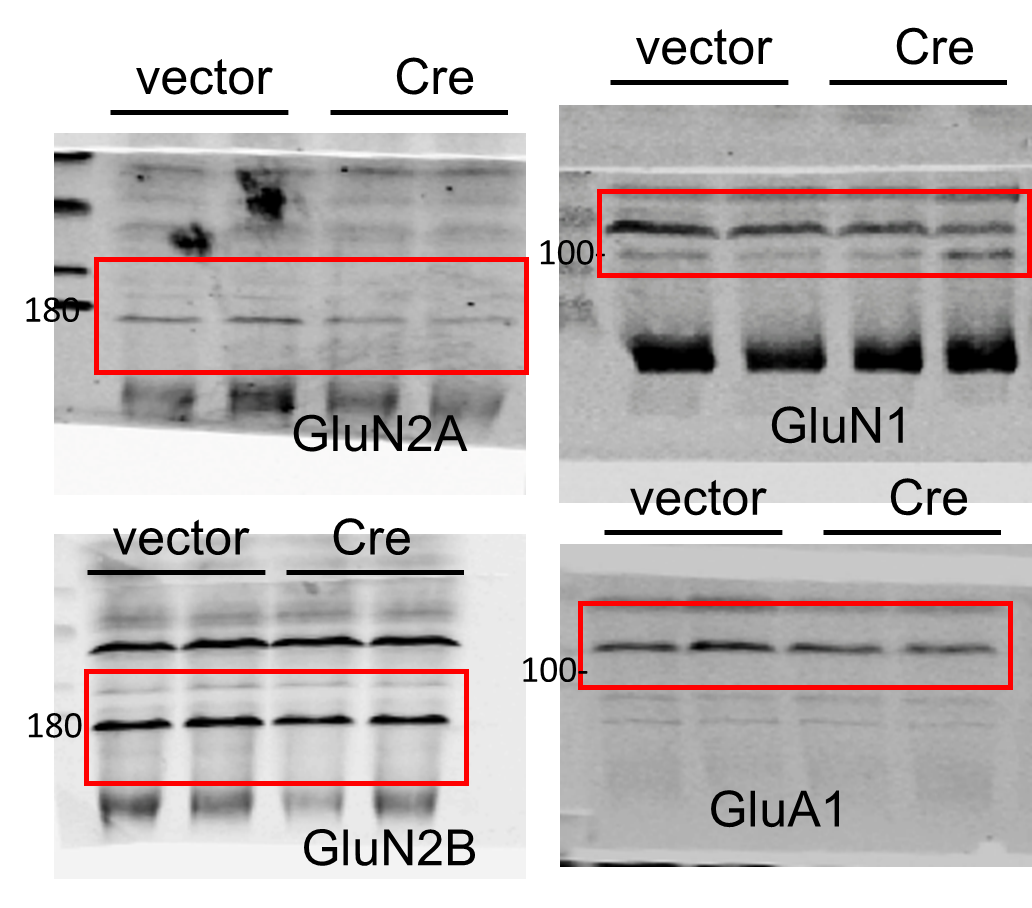


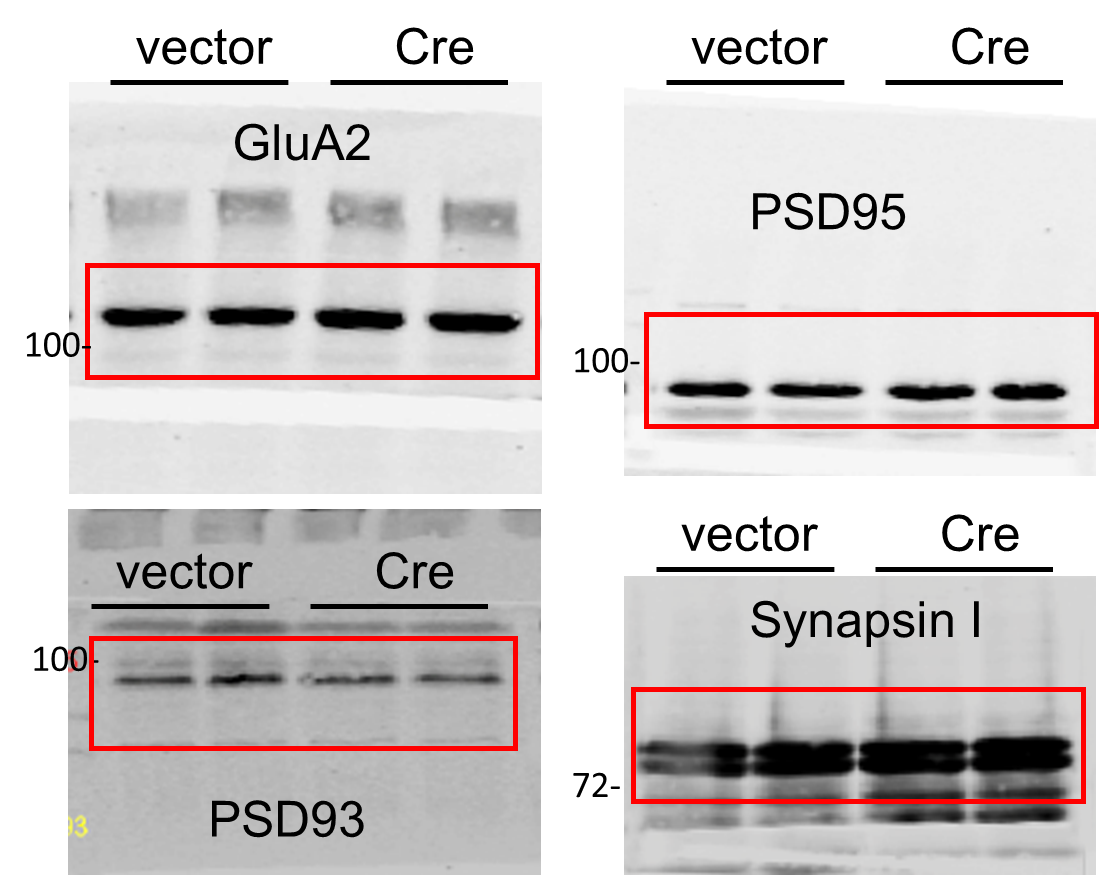


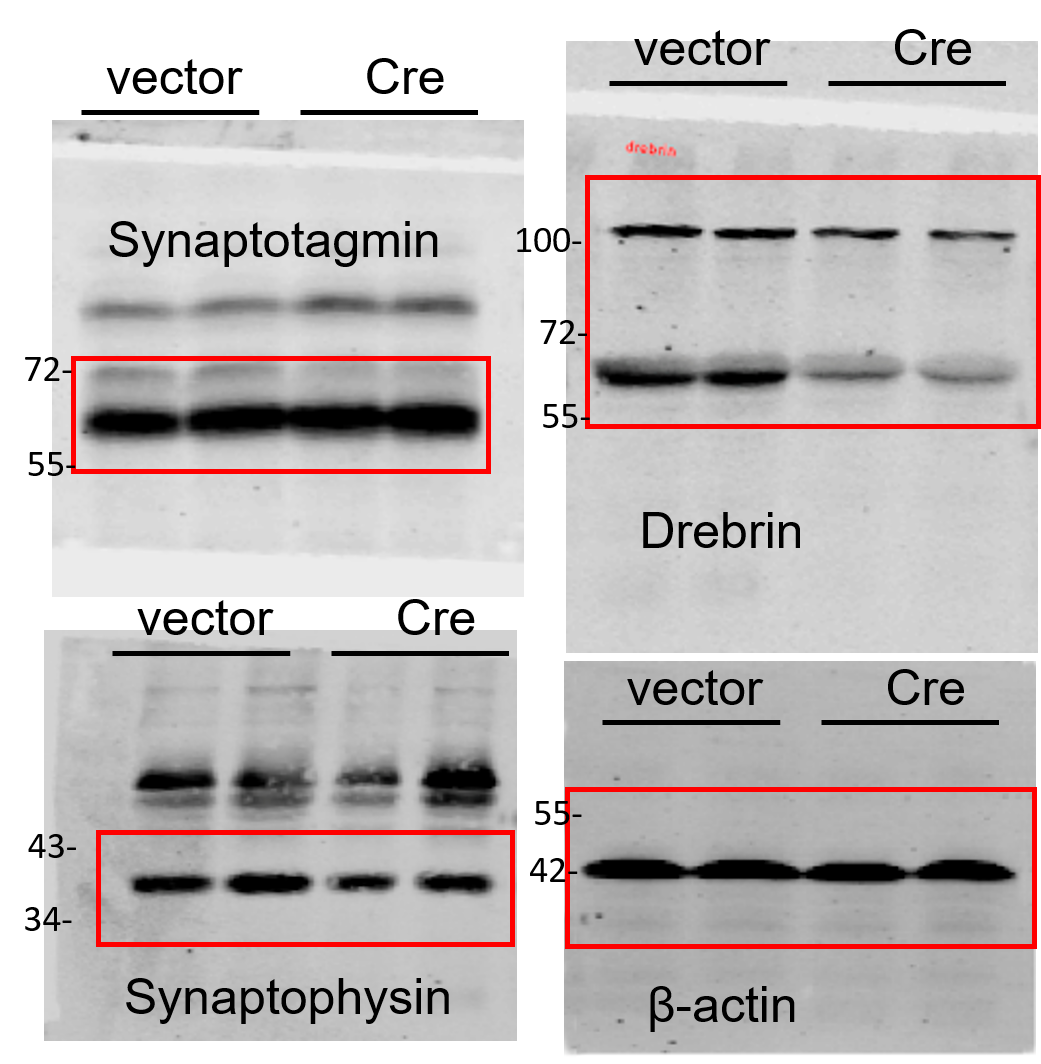


**Supplementary Figure 5. Full-length blots of Figure 4c**


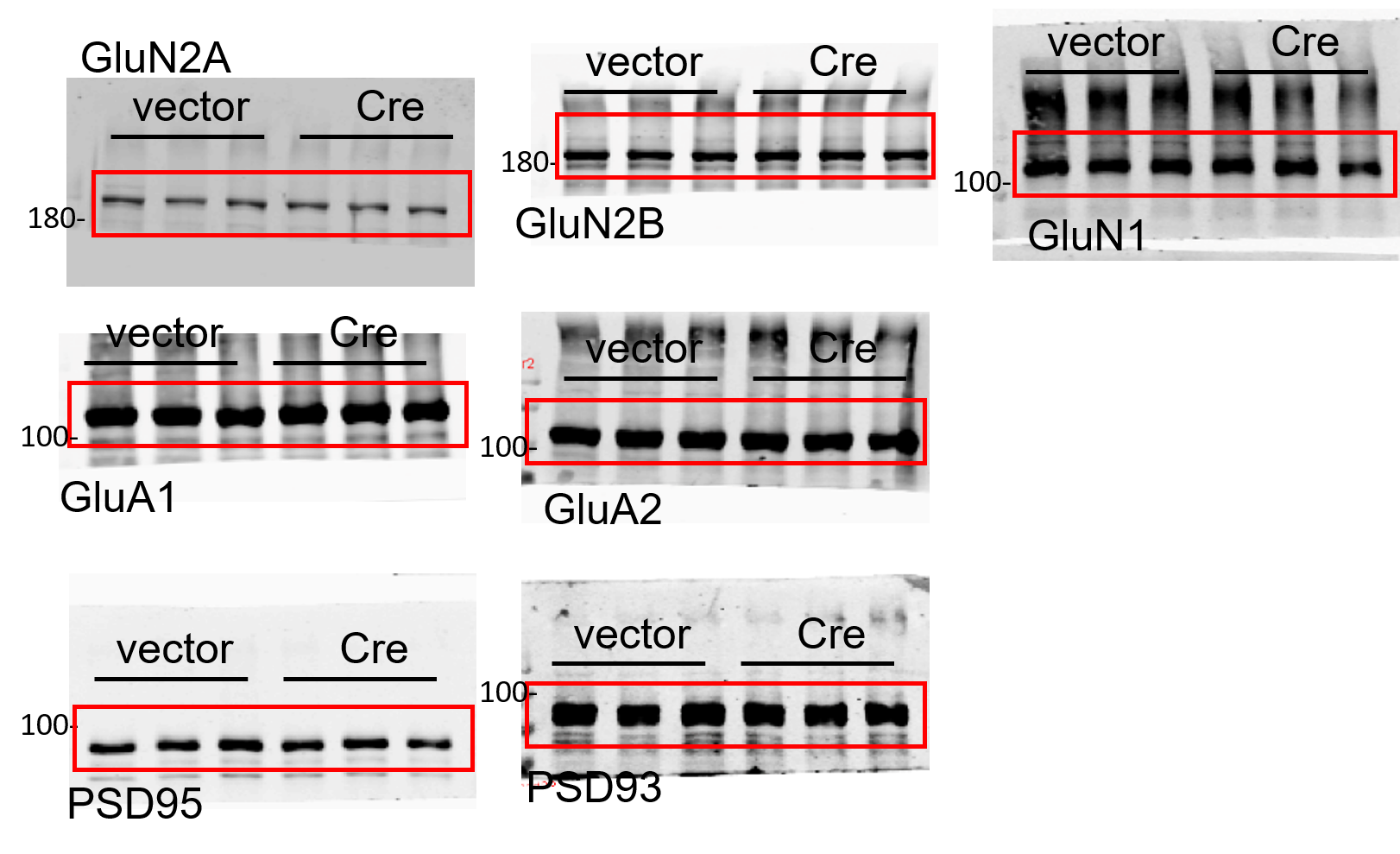


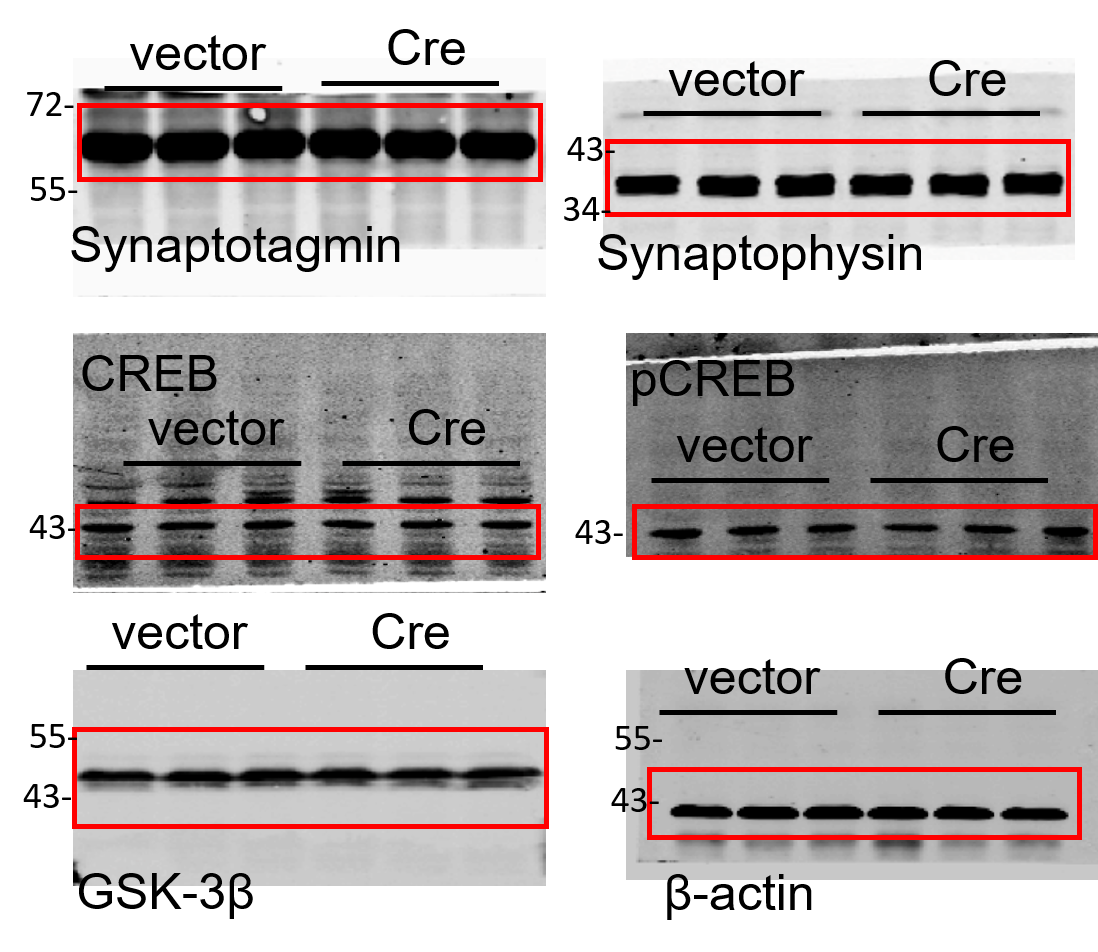


**Supplementary Figure 6. Full-length blots of Figure 6a**


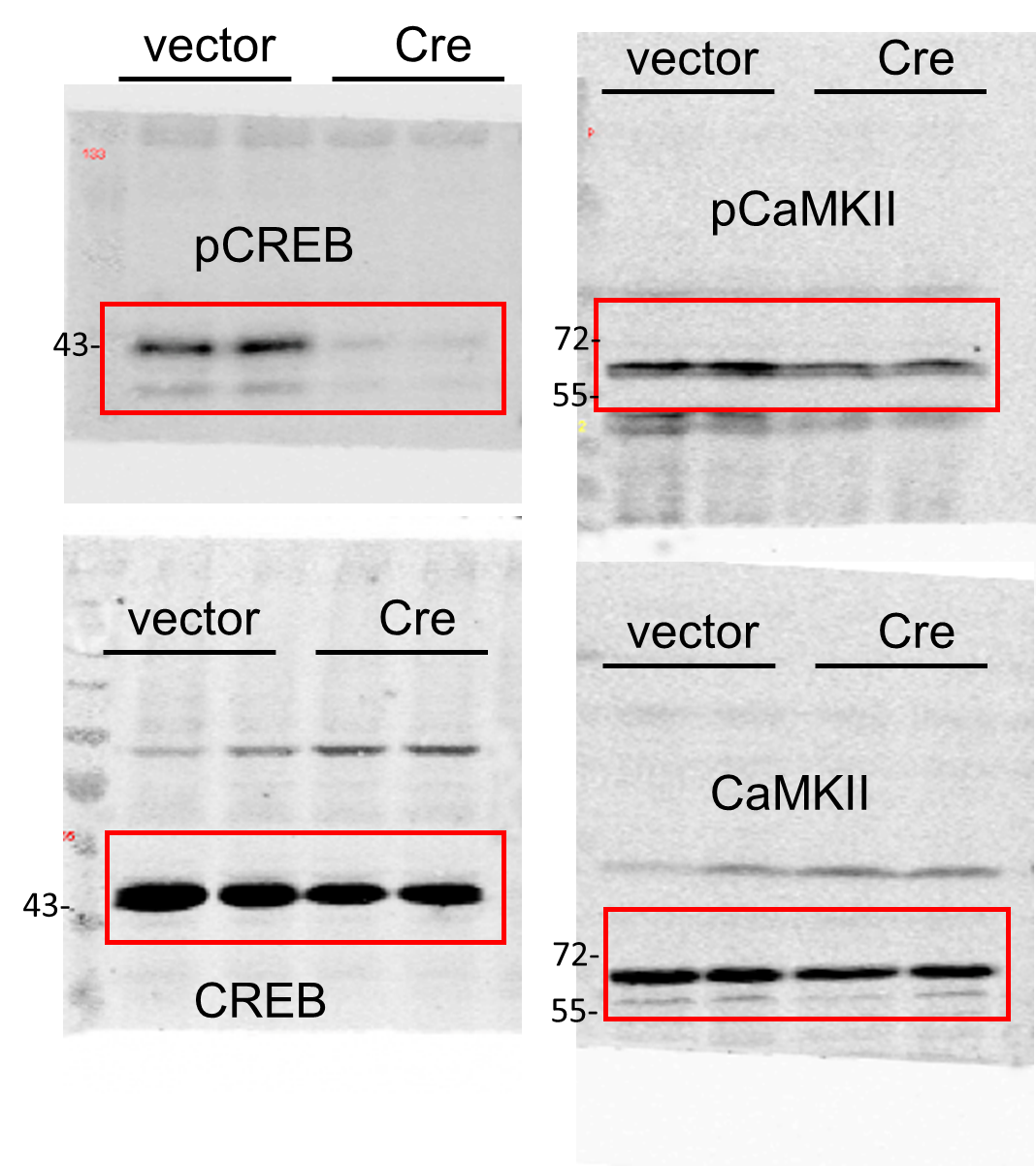


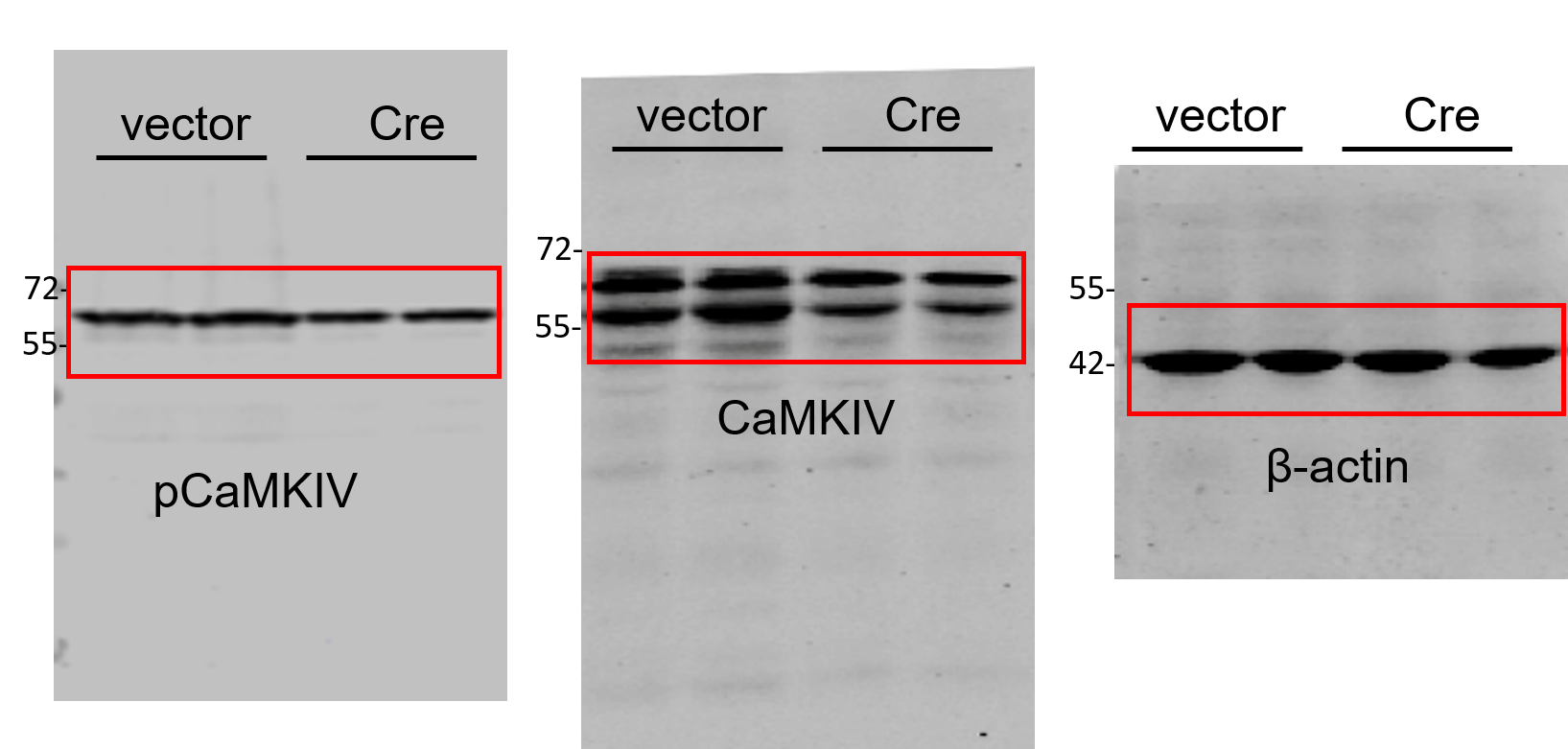

Supplement: Supplementary file 1 — Supplementary Information [file 41598_2017_6173_MOESM1_ESM.doc]
